# Severe Hypophosphatemia in Alcohol-Induced Acute Pancreatitis: A Case Report

**DOI:** 10.7759/cureus.34149

**Published:** 2023-01-24

**Authors:** Usamah Al-Anbagi, Shybin Usman, Abdulrahman Saad, Abdulqadir J Nashwan

**Affiliations:** 1 Department of Internal Medicine, Hamad Medical Corporation, Doha, QAT; 2 Department of Medicine, Ministry of Public Health, Doha, QAT; 3 Department of Nursing, Hamad Medical Corporation, Doha, QAT

**Keywords:** acute pancreatitis, alcohol-induced pancreatitis, electrolyte abnormalities, alcohol, hypophosphatemia, phosphate, pancreatitis

## Abstract

Mild hypophosphatemia can be reported in some cases of acute pancreatitis, especially in alcohol-induced pancreatitis (AIP), but severe hypophosphatemia (≤0.33 mmol/L) is rarely reported, even in cases with AIP. Here, we have a case report of a 43-year-old male with alcohol-related pancreatitis associated with a critical value of hypophosphatemia. We urge clinicians to consider undertaking serial monitoring of the serum phosphate levels of any patient admitted with acute pancreatitis, especially when alcohol is involved.

## Introduction

Acute pancreatitis is not listed among the causes of severe (critical value) hypophosphatemia [1]. A few major conditions can be associated with symptomatic hypophosphatemia. The conditions are chronic alcoholism, intravenous hyperalimentation without phosphate supplementation, urinary phosphate-wasting syndromes, and the treatment of diabetic ketoacidosis [2]. Four common mechanisms lead to hypophosphatemia: the redistribution of phosphate from the extracellular fluid into the cells, the decrease in phosphate absorption from the intestine, the removal of phosphate by kidney replacement therapies, and increased urinary phosphate excretion through the urinary system [2].

This article has been posted as a preprint on Authorea [3].

## Case presentation

A 43-year-old gentleman presented to the emergency department of our hospital with severe periumbilical pain, fever, and recurrent vomiting for the past two days. He was a smoker and imbibes alcohol occasionally during the weekends, with no known comorbidities or regular medications. He had consumed alcohol (500 ml whiskey) four days before presenting to the hospital. He had mild diffuse abdominal discomfort and nausea after consuming alcohol. The symptoms worsened over the next few days until he presented to the hospital with severe symptoms. His fever was low-grade. The abdominal pain was limited to the periumbilical area, dull in nature, and radiated to the back. Vomiting was non-projectile. The initial vomitus contained food particles, while later, he vomited scanty amounts of green fluid only. He claimed to have vomited 10-13 times per day before the presentation. He had no chills, rigors, dysuria, diarrhea, cough, breathlessness, chest pain, or throat pain.

On physical examination, he was tachycardic (heart rate: 123 beats per minute) and had high blood pressure (144/103 mmHg). His respiratory rate was 21 breaths per minute, and oxygen saturation (SpO2) was 96% on room air. He was afebrile (temperature: 37.1°C). His weight was 64 kg. He had no pallor, icterus, cyanosis, clubbing, or edema. His chest was clear with no added or adventitious sounds. No abnormality was detected in the cardiovascular system.

Abdominal examination revealed mild upper abdominal tenderness only. No visceromegaly, guarding, or evidence of free fluid was detected. Examination of the nervous system was also within normal limits.

The findings of the imaging tests done as part of management were as follows: chest X-ray (posteroanterior view) showed bilateral accentuation of vascular markings in the lung fields with no obvious pneumonic consolidation. The mediastinal and cardiac shadows were normal. There was no pleural effusion. The thoracic cage was normal. The abdomen X-ray showed a nonspecific bowel gas pattern and no air-fluid levels or free intraperitoneal gas.

**Figure 1:**
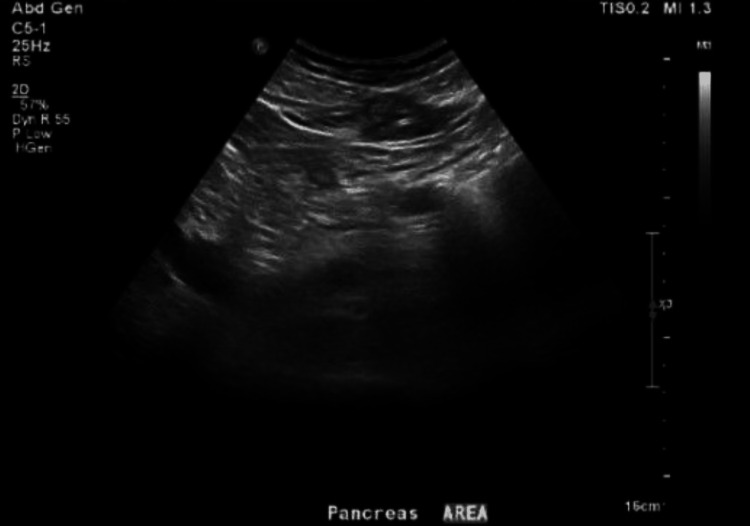
Abdominal ultrasound showed only fatty liver and the pancreas was obscured by bowel gas

Abdominal ultrasound showed only fatty liver. But the pancreas was obscured by bowel gas (Figure 1). There were no sonographic features of acute cholecystitis or cholelithiasis. The liver appeared normal in size, measuring 14.9 cm, and showed increased echogenicity. No focal lesion or intrahepatic biliary radicle dilatation was seen. The common bile duct (CBD) was not dilated and measured 3 mm in diameter. The gallbladder was well distended without wall thickening, cholelithiasis, or pericholecystic fluid.

**Figure 2:**
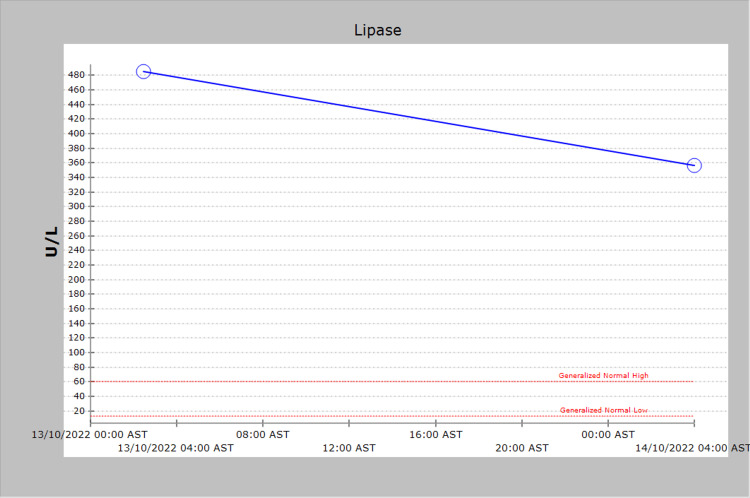
Serum lipase levels

The patient was diagnosed with acute alcoholic pancreatitis based on the history, examination, and high lipase level (typical abdominal pain and high lipase level) (Figure 2).

**Figure 3:**
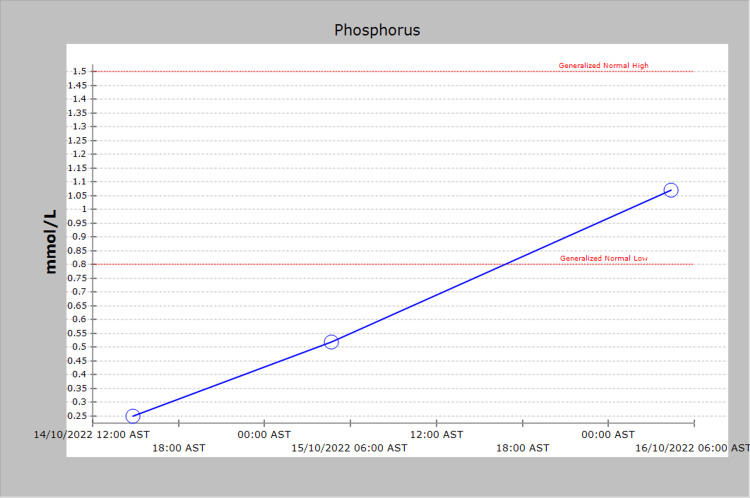
Serum phosphorus levels

The patient received two liters of normal saline as a bolus fluid followed by another two liters of 5% dextrose in normal saline (DNS) as a continuous infusion at a rate of 125 ml/hour. Phosphate replacement was started initially with 20 mmol of sodium glycerophosphate as a slow intravenous infusion over eight hours, followed by an oral potassium/sodium phosphate tablet (Neutra®) at a dose of 250 mg three times a day (Figure 3).

This led to progressive correction of phosphate levels. He was discharged on the fourth day of admission with the serum phosphate level and other laboratory tests returned to normal values (Table 1).

**Table 1:** Laboratory investigations HbA1c: hemoglobin A1C; AST: aspartate aminotransferase; ALT: alanine transaminase; TSH: thyroid-stimulating hormone; FT3: free triiodothyronine.

Laboratory parameters	On admission	2nd day of admission	Upon discharge	Reference values
Serum phosphate (mmol/L)	Not done	0.25	1.07	0.8-1.5
Total leukocytes	13	6.2	6.8	6.2 x 10^3/uL
Hematocrit	46	35.4	41.2	40-50%
Serum potassium (mmol/L)	4.4	3.5	4	3.5-5.3
Serum sodium (mmol/L)	132	136	138	133-146
Serum calcium (mmol/L)	Not done	2.3	2.55	2.2-2.6
Serum magnesium (mmol/L)	Not done	0.88	0.82	0.7-1
Serum urea (mmol/L)	7.5	5	2.1	2.5-7.8
Serum creatinine (umol/L)	140	79	49	62-106
Serum glucose (mmol/L)	6.5	6.2	7.1	<11.1
HbA1c	5.7%			<5.7%
Serum albumin (gm/L)	53	37	43	35-50
Serum total protein (gm/L)	94	67	62	60-80
Serum lipase (U/L)	485	356	Not done	13-60
Lactate (mmol/L)	1.3	1.5	1.6	0.5-2.2
AST (IU/L)	48	42	70	0-41
ALT (IU/L)	44	32	50	0-41
Alkaline phosphatase (U/L)	100	67	64	40–129
TSH (mIU/L)	1.52			0.3-4.2
FT3 (pmol/L)	17.9			11-23.3
Serum total bilirubin (mg/dl)	34	21	17	0-21
Serum chloride (mmol/L)	84	100	96	95-108
Serum bicarbonate (mmol/L)	10	22	26	22-29
24-hour urine phosphorous (mg/24 hours)	Not done	<1.1		
Serum triglycerides (mmol/L)	3.4	Not done	1.2	<1.7

## Discussion

Severe (critical value) hypophosphatemia is rarely reported as one of the complications in acute pancreatitis and has been attributed mainly to alcohol abuse [4-6]. Patients with alcohol use disorder are at risk of severe hypophosphatemia, particularly if requiring hospitalization. This consequence is mostly due to the underlying chronic phosphate depletion from the body, complicated by acute shifts of phosphate from the extracellular into the intracellular compartment [5].

Hypophosphatemia can lead to various symptoms and clinical manifestations [5]. The manifestations largely depend upon the chronicity and severity of the phosphate depletion. It includes muscle pain, bone pain, muscle weakness, altered mental status, numbness, seizure, and even coma in severe life-threatening hypophosphatemia [5].

Although phosphate depletion is common in hospitalized patients with alcohol use disorder, the drop in the phosphate level (to less than 0.32 mmol/L) may not become obvious and prominent until 12-36 hours post-admission and is attributed to the movement of phosphate from the extracellular into the intracellular space [7,8].

Two factors may contribute to this shift. The first is intravenous dextrose-containing solutions that are usually administered in such situations. Glucose in the blood stimulates the release of insulin from the pancreas, which promotes phosphate uptake by the cells as phosphorylated glucose intermediates [8]. If intravenous dextrose infusion is discontinued, a movement of phosphate into cells may still occur because of refeeding-induced endogenous insulin release [9,10]. Even a small amount of 5% dextrose can lead to hypophosphatemia [10-12].

The second factor is acute respiratory alkalosis induced by hyperventilation as a complication of alcohol withdrawal. The increase in extracellular pH (potential of hydrogen) produces a similar change in intracellular pH as carbon dioxide can rapidly diffuse across cell membranes. The ensuing increase in intracellular pH (alkalosis) stimulates intracellular phosphofructokinase, leading to an increase in glycolysis and causing the movement of phosphate into cells [13].

Our patient had one of the above-mentioned factors, which was the dextrose infusion he received. This likely contributed to the drastic decrease in his serum phosphate level. Hypophosphatemic patients with alcohol use disorder may present with skeletal muscle myopathy due to both the effects of alcohol and phosphate depletion. And they also are at risk of developing rhabdomyolysis [9].

## Conclusions

Severe (critical value) hypophosphatemia is rarely reported as one of the complications in acute pancreatitis and has been attributed mainly to alcohol abuse, particularly if requiring hospitalization. Two factors may contribute to phosphate depletion in such a situation, the first is intravenous dextrose-containing solutions, and the second is acute respiratory alkalosis due to alcohol withdrawal. Here, we report a case of severe (critical value) hypophosphatemia associated with alcohol-induced acute pancreatitis. We highly suggest undertaking serial monitoring of the serum phosphate levels of any patient admitted with acute pancreatitis, especially when related to alcohol.
